# Pyridoxal Isonicotinoyl Hydrazone Improves Neurological Recovery by Attenuating Ferroptosis and Inflammation in Cerebral Hemorrhagic Mice

**DOI:** 10.1155/2021/9916328

**Published:** 2021-09-08

**Authors:** Hengli Zhang, Min Wen, Jiayu Chen, Chaojie Yao, Xiao Lin, Zhongxiao Lin, Junnan Ru, Qichuan Zhuge, Su Yang

**Affiliations:** ^1^Zhejiang Provincial Key Laboratory of Aging and Neurological Disorder Research, Department of Neurosurgery, The First Affiliated Hospital of Wenzhou Medical University, Wenzhou 325000, China; ^2^Department of Neurosurgery, Guangzhou First People's Hospital, School of Medicine, South China University of Technology, Guangzhou 510000, China

## Abstract

Ferroptosis and inflammation induced by cerebral hemorrhage result in an excessive inflammatory response and irreversible neuronal injury. Alleviating ferroptosis might be an effective way to prevent neuroinflammatory injury and promote neural functional recovery. Pyridoxal isonicotinoyl hydrazine (PIH), a lipophilic iron-chelating agent, has been reported to reduce excess iron-induced cytotoxicity. However, whether PIH could ameliorate the effects of hemorrhagic stroke is not completely understood. In the present study, the preventive effects of PIH in an intracerebral hemorrhage (ICH) mouse model were investigated. Neurological score, rotarod test, and immunofluorescence around the hematoma were assessed to evaluate the effects of PIH on hemorrhagic injury. The involvement of ferroptosis and inflammation was also examined in vitro to explore the underlying mechanism. Results showed that administration of PIH prevented neuronal cell death and reduced lipid peroxidation in Erastin-treated PC-12 cells. In vivo, mice treated with PIH after ICH attenuated neurological deficit scores. Additionally, we found PIH reduced ROS production, iron accumulation, and lipid peroxidation around the hematoma peripheral tissue. Meanwhile, ICH mice treated with PIH showed an upregulation of the key ferroptosis enzyme, glutathione peroxidase 4, and downregulation of cyclooxygenase-2. Moreover, PIH administration inhibited proinflammatory polarization and reduced interleukin-1 beta and tumor necrosis factor alpha in ICH mice. Collectively, these results demonstrated that PIH protects mice against hemorrhage stroke, which was associated with mitigation of inflammation and ferroptosis.

## 1. Introduction

Intracerebral hemorrhage (ICH) is a destructive subtype of stroke that results in high mortality and morbidity, which has few viable therapeutic options to intervene against primary and subsequent secondary brain injury (SBI). To date, the main clinical treatment is to remove the hematoma through neurosurgery to reduce mechanical compression [1, 2], with no effective treatment for subsequent SBI [3]. ICH involves complex pathophysiological mechanisms, with emerging evidence indicating the involvement of ferroptosis in the pathological mechanism of SBI [4–6]; therefore, novel therapies against ICH-induced ferroptosis may have tremendous therapeutic benefits.

Previous studies have indicated that neuroinflammation and ferroptosis have significant roles in SBI [7]. Ferroptosis is a recently discovered form of nonapoptotic cell death identified by Stockwell et al. in 2012. It is characterized by excessive iron-dependent accumulation of lipid peroxide, accompanied by the production of a large number of reactive oxygen species (ROS) [8]. ROS, in turn, is regulated by glutathione peroxidase 4 (GPX4), which can scavenge lipid peroxides at the expense of glutathione (GSH) [9]. Recent studies have confirmed that hemorrhage-induced red blood cell infiltration and its subsequent hemoglobin degradation initiate neural cell ferroptosis and causes irreversible damage to neurons [7, 10]. Importantly, ferroptosis is coupled with neuroinflammation by triggering the release of damage-associated molecular patterns, immunogenic lipid [11], and proinflammatory cytokines from activated immune cells [12, 13], while activated immune cells, like microglia and peripheral macrophage, release inflammatory cytokines, which simultaneously promote ROS production for ulterior stimulation. Therefore, targeting redox imbalance and inflammation may be a promising therapeutic strategy in neurological diseases involving severe neuronal death and neuroinflammation.

An iron chelator agent is a promising ferroptosis therapy. Desferrioxamine (DFO), a common iron-complexing agent used in the clinic, has been proven to inhibit ferroptosis; however, its low permeability and short plasma half-life limit its clinical application [14, 15]. Thus, the necessity to find alternative iron-complexing agents is evident. Pyridoxal isonicotinoyl hydrazine (PIH) is a promising lipophilic iron-complexing agent with the advantages of high iron chelation efficiency, high permeability, and low cytotoxicity [16–19]. In animal models, after PIH treatment, the complex formed by PIH and iron gradually accumulates in rat bile and is mainly excreted through the digestive system [20]. Previous studies showed that PIH and its analogues exhibited properties such as antitumor proliferation, cell oxidative damage prevention, excessive iron overload reduction, and myocardial injury amelioration [21–23]. However, little is known about the PIH-related protective mechanism associated with ferroptosis and anti-inflammation inhibition in ICH. In this study, we investigated the neuroprotective effect of PIH in ICH and explored the involvement of the PIH-mediated effect on ferroptosis and inflammation to explore the underlying inhibition-associated mechanisms.

## 2. Materials and Methods

### 2.1. Animals and Groups

Adult male C57BL/6 mice (20–25 g) were purchased from the Shanghai Slaccas Experimental Animal Limited Liability Company (Shanghai, China). All mice are raised in a standardized animal care center, under a 12 h light-dark cycle with free access to food and water. All procedures were approved by the Ethics Committee of Wenzhou Medical University (Wenzhou, China) and carried out under its supervision. Mice were randomly divided into four groups: the sham group, ICH group, ICH treated with DMSO group (ICH+DMSO), and ICH treated with PIH (MCE, USA) group (ICH+PIH). PIH was dissolved in DMSO and injected intraperitoneally (25 mg/kg) at 2, 12, 24, and 48 h after ICH, as previously described [24].

### 2.2. ICH Model

Collagenase was used to construct the ICH mouse model [25]. Briefly, mice were anesthetized by inhalation with 8% isoflurane and maintained using a face mask with 4% isoflurane in a 5 l/min oxygen flow. The mice were then fixed on a stereotactic head frame (Kopf Instruments, Tujunga, CA, USA), and a 1 cm incision was made in the center of the scalp, after which the skin was removed and the skull exposed for suitable bone window drilling. To induce ICH, 1 *μ*l collagenase VII-S (Sigma, 0.3 U) was injected, 0.2 mm anterior to and 2.0 mm right to the bregma, at a depth of 3.7 mm and rate of 2 *μ*l/min (right striatum). The microsyringe was not moved for 10 min after injection, after which it was taken out at a rate of 1 mm/min. The sham operation group received the operation, but with normal saline injection instead.

### 2.3. Cell Culture

The PC12 cell line was purchased from the American Type Culture Collection (ATCC, USA). All cells were cultured in Dulbecco's minimal essential medium (GE Healthcare Life Science, Pittsburgh, PA, USA) with 10% fetal bovine serum (Gibco, Thermo Fisher Scientific, Waltham, MA, USA), 100 U/ml penicillin, and 100 *μ*g/ml streptomycin (Gibco Life Technologies, Darmstadt, Germany) under standard conditions. Erastin and PIH were obtained from MedChemExpress (MCE). For drug administration, PIH (10 *μ*M) and Erastin (5 *μ*M) were added concurrently for 24 h prior to cell lysis or imaging.

### 2.4. MTT Assay

Cell viability was measured with MTT (Beyotime, Shanghai, China) assays. In brief, cells were cultured in a 96-well plate containing 0.5% MTT medium for 4 h. After removing the supernatant, the insoluble formazan was dissolved in 150 *μ*l DMSO. The absorbance at a wavelength of 540 nm was used to calculate cell viability.

### 2.5. Propidium Iodide (PI) Staining

Briefly, after drug treatment, PC12 cells were stained with 1 *μ*g/ml PI staining reagent (Beyotime, Shanghai, China) for 5 min, then fixed with 4% paraformaldehyde (Solarbio, Beijing, China) for 20 min. A fluorescence microscope (DMi8, Leica, Germany) was used to calculate fluorescence intensity.

### 2.6. Iron Content Determination

Iron content was determined using the iron colorimetric assay kit (Prlygen, Beijing, China) and tissue iron assay kit (Nanjing Jiancheng Bioengineering Institute, Nanjing, China). To detect cell samples, 1 ml cell lysate was added to six-well plates, which were then placed on a shaker for approximately 2 h. The cell suspension was then mixed with the detection reagent for 1 h. After that, 30 *μ*l iron ion detection solution was added, and the absorbance at 550 nm was detected to calculate the iron concentration. To detect the iron content in tissue, samples were homogenized and supernatant was taken after centrifugation at 2000 *g* for 10 min, and the absorbance was determined of each group at 520 nm according to the manufacturer's instructions.

### 2.7. ROS Production Assay

Cellular ROS levels were determined using 2′,7′-dichlorofluorescin diacetate (DCFH; Beyotime, Shanghai, China). Briefly, cells were incubated with DCFH (10 *μ*m) for 30 min at 37°C. The fluorescence intensity of the cells was then measured by flow cytometry (CytoFLEX, Beckman, USA). To determine ROS levels in tissue samples, the BB cell Probe™O12 reactive oxygen probe (BestBio, Shanghai, China) was used to detect the ROS content according to the manufacturer's instructions. Briefly, tissue samples were homogenized and centrifuged (4°C, 12000 *g*, 25 min). After that, the supernatant was mixed with 10 *μ*l O12 probe for 30 min at 37°C in the dark. Subsequently, the fluorescence value was detected at an excitation wavelength of 488 nm and an emission wavelength of 540 nm in the enzyme labeling instrument (Thermo Fisher Scientific, Waltham, MA, USA). The mean immunofluorescence intensity of each group was calculated for comparison.

### 2.8. Quantitative Determination of Malondialdehyde (MDA)

MDA content was determined using a lipid peroxidation MDA assay kit (Solarbio, Beijing, China) according to the manufacturer's instructions, while protein concentration was detected by using a BCA protein assay kit (Thermo Fisher Scientific, USA) according to the manufacturer's instructions. Briefly, cells or tissues were homogenized and centrifuged. The supernatant was then mixed with 200 *μ*l MDA working reagent and boiled for 30 min. The absorbance at 450 nm, 532 nm, and 600 nm was detected. Results are expressed as nanomolar concentration per microgram of total protein.

### 2.9. Western Blot Analysis

Brain tissue was removed from euthanized mice and immediately homogenized in RIPA buffer plus with protease inhibitor mixture (PMSF : RIPA = 1 : 100). After homogenization, the protein concentration was determined using a BCA protein assay kit (Thermo Fisher Scientific, USA). Protein was then loaded onto a 12% SDS resolving gel and subject to SDS-PAGE. Proteins were then transferred onto a polyvinylidene fluoride membrane, and the membrane was blocked with 5% milk at room temperature for 2 h and then incubated with primary antibodies, including COX-2 (1 : 1000, 12282S, CST), GPX-4 (1 : 1000, ab125066, Abcam), and *β*-actin (1 : 1000, ab8227, Abcam) overnight at 4°C. The membrane was then incubated with the secondary antibody (1 : 5000, ab205718, Abcam) at room temperature for 1 h, and the bands were visualized using ECL Reagent (Advansta, California, USA) and analyzed with ImageJ.

### 2.10. Immunofluorescence

Brain sections were fixed with 4% paraformaldehyde and then incubated in PBST (0.4% triton in PBS) containing 5% bovine serum albumin solution (Sigma-Aldrich, USA) for 30 min at 37°C to rupture the cell membrane and block nonspecific staining. After that, sections were incubated with primary antibodies against NeuN (1 : 1000, ab177487, Abcam), IBA1 (1 : 500, ab5076, Abcam), iNOS (1 : 300, ab15323, Abcam), and CD206 (1 : 300, ab8918, Abcam) for 24 h at 4°C followed by incubation with the corresponding secondary antibody at room temperature for 1 h [26]. After washing off unbound antibodies, the nuclei were then stained with DAPI (Biosharp, BL105A) and imaged under a fluorescence microscope (DMi8, Leica, Germany). Three brain tissue sections were randomly selected from each mouse for staining, and six microscopic fields were randomly taken around the hematoma in each section. The number of positive cells was calculated and statistically analyzed.

### 2.11. Neurobehavioral Assessment

The modified neurological severity scores (mNSS) test was used to assess neurological deficits. mNSS includes multiple tasks to assess sensation, motor ability, balance, and reflex; a higher score indicates a more severe neurological dysfunction (0–18). The rotarod test was used to evaluate the coordination of the mice [27]. All mice were pretrained for 14 days prior to ICH.

### 2.12. Enzyme-Linked Immunosorbent Assay (ELISA)

ELISA kits (Thermo Fisher Scientific, USA) were used to measure the levels of interleukin-1*β* (IL-1*β*), tumor necrosis factor (TNF-*α*), interleukin-10 (IL-10), and transforming growth factor (TGF-*β*) at 72 h after ICH, following the manufacturer's instructions. Briefly, samples were homogenized in ice-cold PBS and centrifugated at 12,000 *g* for 10 min at 4°C. The supernatant was then measured to determine the levels of IL-1*β*, TNF-*α*, IL-10, and TGF-*β* in accordance with the manufacturer's instructions. Finally, the OD at 450 nm was measured on a microplate reader (Spectra MAX 190; Molecular Devices).

### 2.13. Statistical Analysis

The GraphPad Prism software was used for graphing and statistical analysis. All data are expressed as the mean ± standard deviation. Student's *t*-tests were used for comparisons between two groups, and one-way ANOVA was used for comparison of three or more groups. *P* < 0.05 represents statistical significance.

## 3. Results

### 3.1. PIH Prevents Excess Iron Accumulation and Lipid Peroxidation Induced by Erastin Treatment in PC12

**Figure 1:**
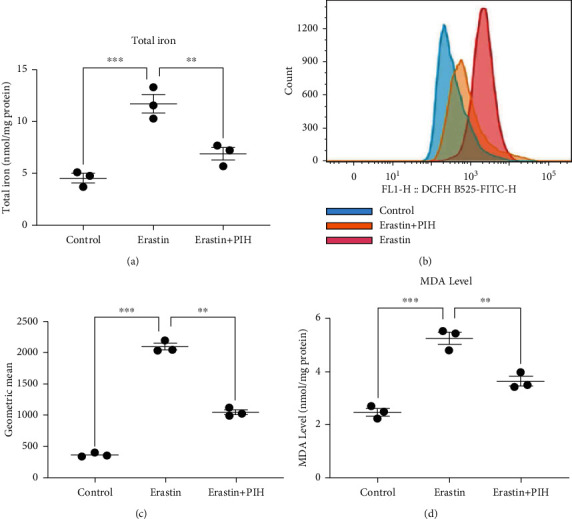
PIH inhibited Erastin-induced iron accumulation, MDA level, and ROS production in PC12. (a) Total iron concentration was detected by iron colorimetric assay kit (*F* = 30.98). (b, c) Cellular ROS levels were evaluated by flow cytometry using DCFH-DA probe (*F* = 555.6). (d) MDA activity was measured with lipid peroxidation MDA assay kit (*F* = 59.61, *n* =3). The data are presented as means ± SD. ^∗∗^*P* < 0.01, ^∗∗∗^*P* < 0.001; ns: not significant.

Intracellular iron is an essential factor in the mechanism of ferroptosis, and the initiation of lipid peroxidation is triggered by a series of oxidizing reactions, which require transition metals such as iron. Here, we used Erastin to induce ferroptosis in vitro [8]. After confirming the toxicity of Erastin and PIH on cell viability (Figure S1), we found that the Erastin group gained massive iron accumulation (11.77 ± 1.37 nmol/mg protein), which was more obvious than that in the sham group (4.53 ± 0.75 nmol/mg protein), whereas the iron concentration in the PIH group was markedly decreased (6.87 ± 1.01 nmol/mg protein, *P* < 0.01, Figure 1(a)). We also explored ROS production and the lipid peroxide accumulation upon Erastin treatment. Flow cytometry data showed that Erastin significantly stimulated the production of ROS, while PIH treatment significantly reduced the level of ROS (Figures 1(b) and 1(c)). As the most important end product of lipid peroxidation, MDA accumulation induced by Erastin was also decreased after PIH administration, from approximately 5.15 ± 0.33 to 3.64 ± 0.30 nmol/mg protein (Figure 1(d)).

### 3.2. PIH Prevents Ferroptosis through Induction of GPX4 and COX-2 Expression

**Figure 2:**
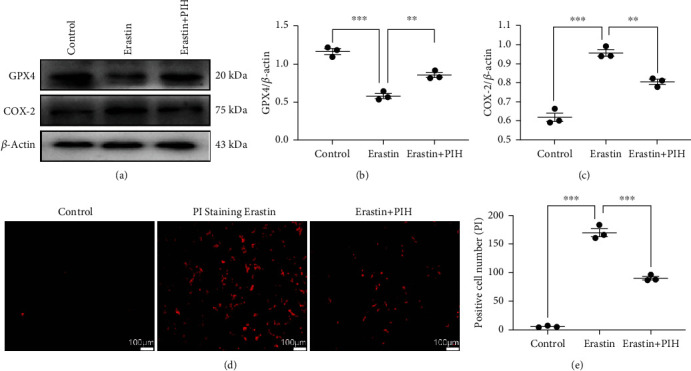
The antiferroptosis activity of PIH in Erastin treated PC12 cells. (a–c) The ferroptosis markers GPX4 and COX-2 were determined through western blotting assay (*F*_b_ = 77.83, *F*_c_ = 92.69). (d, e) PI staining was applied to evaluate neural cell death after Erastin treatment (*F* = 343.3, *n* = 3). The data are presented as means ± SD. ^∗∗^*P* < 0.01, ^∗∗∗^*P* < 0.001; ns: not significant.

Gpx4 is a vital antioxidant enzyme, and COX-2 is the gene product of PTGS2, which can be used as a biomarker of ferroptosis. Western blotting analysis showed decrease expression of GPX4 and increased expression and COX-2 after Erastin treatment, indicating induction of ferroptosis after Erastin administration (Figures 2(a)–2(c)). Administration of PIH significantly promoted GPX4 expression and inhibited COX-2 induction, suggesting the antiferroptosis function of PIH in Erastin model.

PI staining was used to label dead cells. The fluorescence results showed that Erastin caused large amounts of cell death (Figures 2(d) and 2(e)), which was consistent with the results of MTT assays, while the PIH group (85.33 ± 6.51) exhibited decreased neural death compared to the Erastin group (164.70 ± 10.07). These results demonstrated that PIH could inhibit Erastin-induced ferroptosis and prevent neural cell death.

### 3.3. PIH Improves Neurological Recovery after ICH

**Figure 3:**
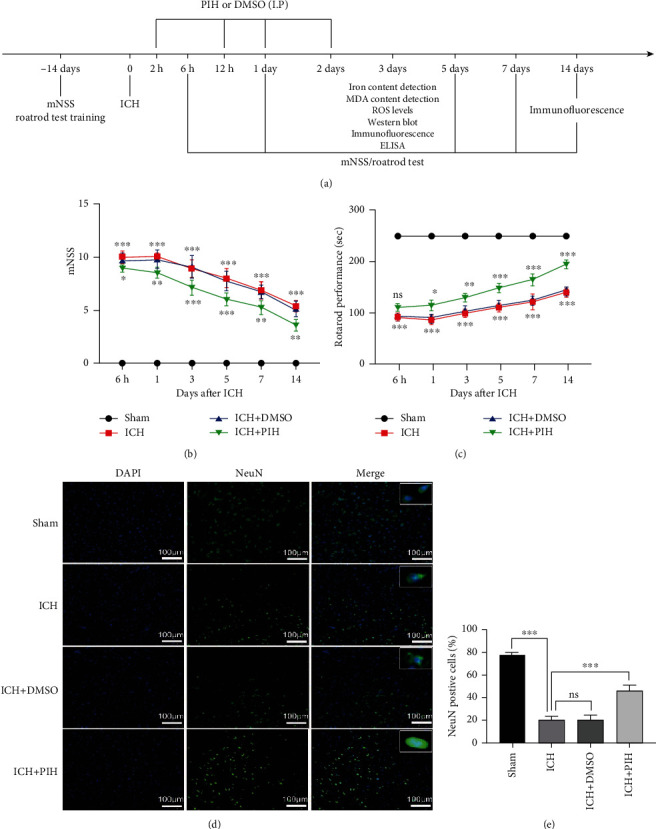
PIH reduced neuronal death and improved neurological function recovery in ICH mice. (a) Experimental design time axis. (b) Modified neurological severity scores (mNSS) and (c) rotarod test were performed to evaluate from day 1 to day 14 post-ICH to evaluate neural function recovery. *n* = 12. (d, e) Immunofluorescence staining was used to detect the survival neurons around the hematoma. NeuN represents neuronal cells (green), DAPI is nucleus (blue) (*F* = 213.3, *n* = 5). The data are expressed as average ± SD, scale = 100 *μ*m. ^∗^*P* < 0.05, ^∗∗^*P* < 0.01, ^∗∗∗^*P* < 0.001; ns: not significant.

To determine whether PIH induced neurological function recovery after hemorrhagic injury, mNSS was applied to evaluate the degree of neuronal damage in ICH mice. As shown in Figures 3(b) and 3(c), the ICH group displayed increased neurological deficit scores compared with the sham group, whereas the PIH group exhibited a significant improvement in neurological scores after day 3, thus suggesting that PIH improved neurological performance in ICH mice. To further investigate the endogenic neurological recovery mechanism, we detected neuronal survival around the hematoma region. The results showed that the number of viable neurons decreased after hemorrhagic injury, while PIH treatment improved neuronal survival (Figures 3(d) and 3(e)), indicating a neuronal protective effect of PIH against hemorrhagic stroke.

### 3.4. Administration of PIH Inhibits Lipid Peroxidation and Ferroptosis in ICH Mice

**Figure 4:**
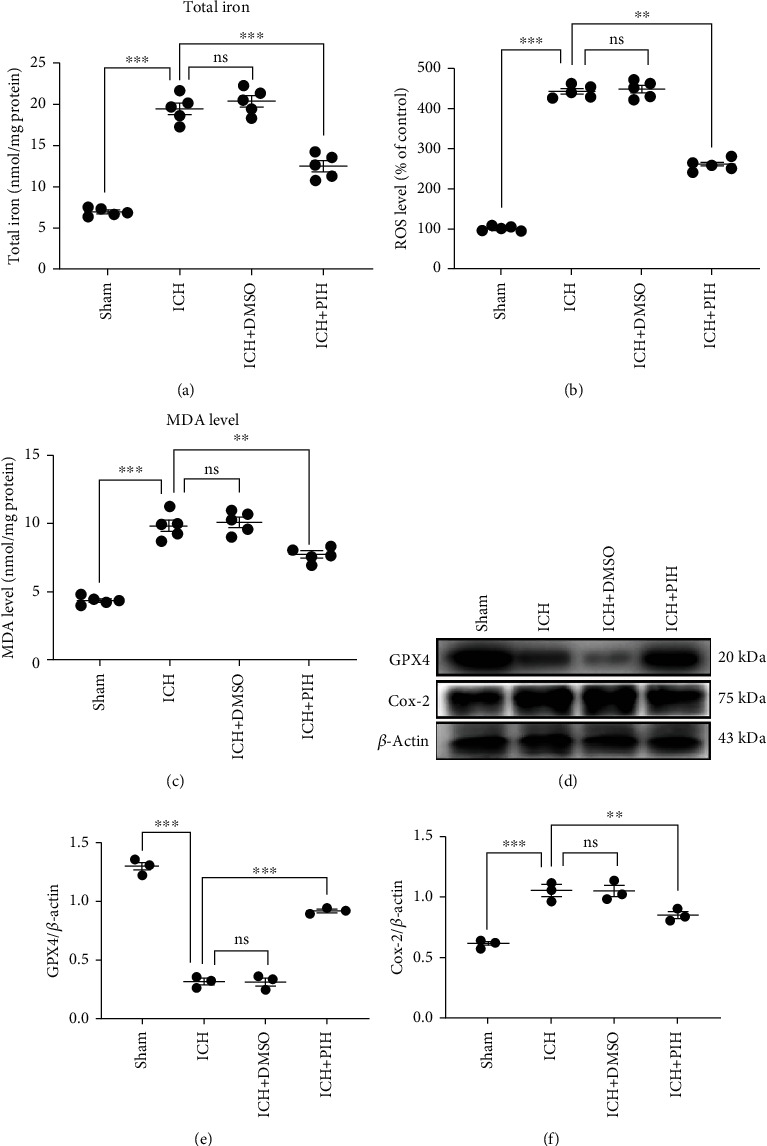
Intraperitoneal injection of PIH significantly inhibited ferroptosis in brain tissue around hematoma. (a) Total iron concentration was detected by tissue iron assay kit (*F* = 106.1). (b) Tissue ROS levels were evaluated by tissue ROS detecting kit using the BB cell Probe™O12 reactive oxygen probe (*F* = 597.4). (c) MDA activity was measured with lipid peroxidation MDA assay kit (*F* = 75.73). (d–f) Western blotting analysis of GPX4 and COX2 expression in ICH mice (*F*_e_ = 257.7, *F*_f_ = 30.06, *n* = 5).. The data are presented as means ± SD. ^∗∗^*P* < 0.01, ^∗∗∗^*P* < 0.001; ns: not significant.

To investigate the antiferroptosis activity of PIH after ICH, we examined the intracellular iron levels, MDA levels, ROS production, and GPX4 and cyclooxygenase-2 (COX-2) expression in ferroptotic neurons within hematoma-adjacent brain tissue. The result showed that the total iron concentration was significantly elevated at 3 days after ICH (Figure 4(a)). Similarly, ROS production and MDA levels were also increased at 3 days after ICH (Figures 4(b) and 4(c)). Western blotting analysis showed decreased GPX4 expression and increased COX-2 expression after ICH. These results indicated the existence of ferroptosis in ICH model mice.

After confirmation of ferroptosis induction after ICH, the antiferroptotic activity of PIH was investigated. The results showed PIH administration remarkably reduced iron accumulation, inhibited ROS production, and reduced MDA levels. Western blotting results showed that GPX4 was significantly increased in the cortex of mice treated with PIH, while COX-2 was restored to physiological levels (Figures 4(d)–4(f)). These data indicated the antiferroptosis efficacy of PIH in the ICH model mice.

### 3.5. PIH Regulated Microglial Polarization in ICH Mice

**Figure 5:**
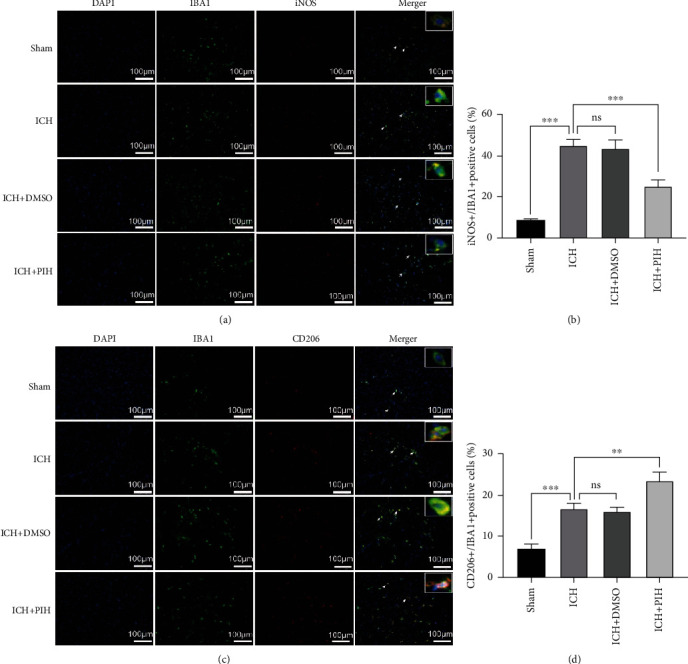
PIH inhibited proinflammatory polarization of microglia in ICH mice. (a, b) Microglia M1 polarization (Iba-1^+^/iNOS^+^) and (c, d) M2 polarization (Iba-1^+^/CD206^+^) were determined by double-immunofluorescence assay (*F*_b_ = 106.3, *F*_d_ = 63.65). Scale = 100 *μ*m. *n* = 5. The data are expressed as average ± SD. ^∗∗^*P* < 0.01, ^∗∗∗^*P* < 0.001; ns: not significant.

To explore the regulatory effect of PIH on microglial polarization, we performed double-immunofluorescence staining to detect M1 polarization (iNOS) and M2 polarization (CD206), microglia cells were labeled with IBA1. Brain sections showed that the number of iNOS^+^/IBA1^+^ cells around the hematoma was significantly increased at 3 days after ICH to approximately 44.87 ± 3.21, while administration of PIH effectively decreased the number to 25.09 ± 3.05 (Figures 5(a) and 5(b)), thus indicating that PIH inhibited microglial M1 polarization. Meanwhile, administration of PIH also increased CD206^+^/IBA1^+^ cells, from approximately 16.66 ± 1.261 to 23.31 ± 2.15 (Figures 5(c) and 5(d)), thus suggesting that PIH promoted microglial M2 polarization after ICH.

### 3.6. PIH Decreased the Expression of Proinflammatory Cytokines after ICH

**Figure 6:**
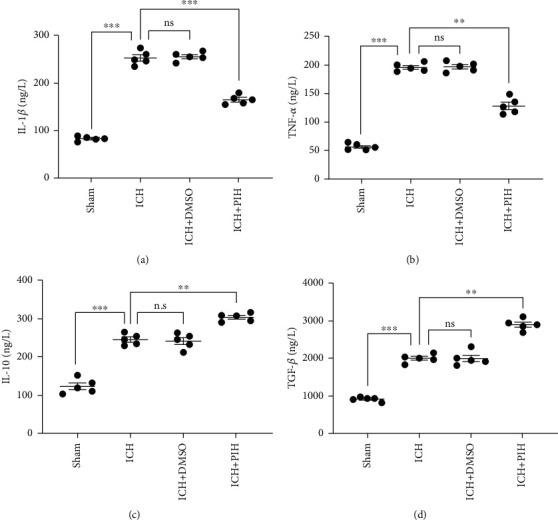
PIH administration affected inflammatory response in brain tissue around hematoma. (a–d) The cytokine expressions of IL-1*β*, TNF-*α*, IL-10, and TGF-*β* were determined by ELISA (*F*_a_ = 320, *F*_b_ = 250.4, *F*_c_ = 106.4, and *F*_d_ = 176, *n* = 5). The data are expressed as average ± SD. ^∗^*P* < 0.05, ^∗∗^*P* < 0.01, ^∗∗∗^*P* < 0.001; ns: not significant.

**Figure 7:**
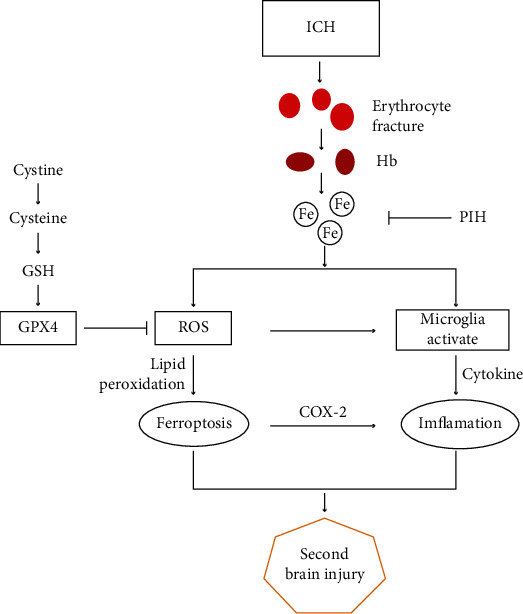
Schematic illustration of the proposed mechanism underlying PIH-mediated protection against ICH. ICH causes ruptured red cells and release of heme, which results in excess iron accumulation. Then excess iron produces ROS through Fenton reaction, which subsequently leads to lipid peroxidation and eventually induces ferroptosis. Iron accumulation and ROS also activate polarization of microglia, leading to the aggravation of inflammatory response. PIH prevents Fenton response through binding iron ions, thereby reduces ROS production, and ultimately inhibits ferroptosis and reduces inflammation. ICH: intracerebral hemorrhage; Hb: hemoglobin; PIH: pyridoxal isonicotinoyl hydrazone; GSH: glutathione; ROS: reactive oxygen species.

Considering the correlation between ferroptosis and inflammation in hemorrhagic stroke, and the fact that PIH reduced M1 polarization, we investigated the possible anti-inflammatory functions of PIH. ELISA results showed the ICH group displayed higher expression levels of IL-1*β*, TNF-*α*, IL-10, and TGF-*β* (Figures 6(a)–6(d)). Notably, following the administration of PIH, a marked decrease in IL-1*β* and TNF-*α* was observed, while the anti-inflammatory cytokines, IL-10 and TGF-*β*, exhibited an increase in the PIH group. The results demonstrated that PIH inhibited the inflammatory response in the hematoma peripheral tissue of ICH mice. Thus, collectively, these data implied PIH might downregulate inflammatory reaction via inhibiting ferroptosis in hemorrhagic stroke mice (Figure 7).

## 4. Discussion

ICH accounts for about 10%–15% of all strokes and has become the leading cause of death in China [28]. Inflammation, oxidative stress, neurotoxicity, and other factors accelerate secondary brain injury and eventually induce irreversible neuronal injury, which seriously affects the prognosis of ICH [29, 30]. Excessive iron and ROS accumulation are considered major contributors to ICH-induced secondary brain injury. After ICH, a large number of red blood cells escape from the blood, rupture, and release hemoglobin, which degrades and produces excess iron ions, initiates lipid peroxidation, and eventually leads to ferroptosis in neurons [4].

As an iron-dependent programmed cell death, ferroptosis exists in the acute stage of ICH and participates in the progression of ICH [31–33]. However, the pathological process of ferroptosis remains elusive. It is reported that large amounts of ferrous ions are transported to neurons by transferrin, which triggers a Fenton reaction and produces a large number of ROS, resulting in neuronal cell death. Currently, methods to monitor ferroptosis rely mainly on measuring ROS production, lipid peroxidation, iron accumulation, and abilities of ferroptosis inhibitors to block cell death [34–36]. According to the previous research results, the ferroptosis inhibitors, ferrostatin-1 and liproxstatin-1, have shown their therapeutic value in animal models of ICH; however, their “ROS inhibitor” roles hinted that they may inhibit other pathways of cell death at the same time [37–39]. It is particularly important to find more selective and safe drugs to promote ICH recovery, and iron-chelating agents may be a potential treatment for ICH-induced ferroptosis.

At present, it has been reported that iron-chelating agents (2,2′-dipyridyl, VK-28) exhibited their neuroprotective effects in reducing brain tissue injury after ICH, suggesting a protective effect of iron-chelating agents against ICH-induced ferroptosis around the hematoma [40, 41]. The most widely used ferroptosis inhibitor (DFO) limits its clinical use due to its high hydrophilicity and strict administration conditions. In this study, we found that PIH, a lipophilic tridentate Fe-chelating agent, inhibited hemorrhagic injury induced by ferroptosis and improved neural functional recovery in an ICH model. Erastin is a classic ferroptosis inducer, which suppresses the glutamate/cystine antiporter and subsequently inhibits cellular cysteine uptake and depletes GSH. GSH functions in maintaining redox balance and defending against oxidative stress. Our results showed that PIH inhibited the accumulation of iron ions, reduced the levels of ROS and lipid peroxidation, decreased GPX4, and increased COX-2 expression, suggesting the antiferroptotic ability of PIH. To investigate its antiferroptotic effect in an ICH model, a collagenase ICH model was established. In agreement with previous results, we found that PIH could effectively reduce the total iron content in the perihematoma tissues and decrease ROS and lipid peroxides content, indicating its antioxidative ability. The increased GPX4 expression and concurrent decreased COX2 expression proved the antiferroptosis capacity of PIH in ICH model mice.

Hemorrhagic injury induces excessive ROS production and lipid oxidation, resulting in neuronal cell death and serious clinical symptoms; and the concomitant inflammation further aggravates these symptoms. Therefore, preventing ferroptosis-induced neuronal cell death would help to ameliorate many of the clinical symptoms associated with ICH. Behavioral test showed that neurological function was recovered after PIH treatment. In addition, although PI staining indicated massive neuronal cell death after ICH, administration of PIH dramatically increased the survival of neurons around the hematoma peripheral tissue and, as a result, restored brain function. These data verified that PIH promoted the neurological functional recovery through inhibiting neuronal cell death.

Neuroinflammation has been considered to play a critical role in secondary brain injury during cerebral hemorrhage. As key immune cells in the nervous system, microglia are rapidly activated after ICH. Activated microglia can differentiate into proinflammatory M1 microglia and produce proinflammatory factors such as IL-1*β*, IL-6, and TNF-*α* to aggravate brain tissue injury. In contrast, microglia can also differentiate into M2 phenotype, which promotes the release of anti-inflammatory factors such as IL-10 and TGF-*β* to accelerate the removal of hematoma and repair of brain tissue. Evidence suggests that promoting microglia polarization to the M2 phenotype may contribute to the recovery of neurological function [42–44]. It is worth noting that in the early stage of ferroptosis, iron is engulfed by and accumulates in microglia, leading to microglia polarization and promoting the formation of M1 microglia [45]. Our results indicated that PIH can effectively promote the transformation of microglia from the M1 to M2 phenotype after ICH. Accordingly, ELISA results suggested that PIH reduces the expression of the proinflammatory cytokines IL-1*β* and TNF-*α* and increases the concentration of the anti-inflammatory factors IL-10 and TGF-*β*, which indicates that PIH elicits its neuroprotective effects through inhibiting inflammatory response.

The correlation between ferroptosis and necroinflammation has yet to be fully elucidated. Increasing evidence has confirmed a direct positive correlation between ferroptosis and inflammation [39, 46, 47]. Unlike the immunologically silent apoptosis, ferroptosis is immunogenic and induces cells to release damage-associated molecular patterns and alarmins, which accelerate cell death and promote inflammation [7, 48]. Excessive iron accumulation and oxidation associated with ferroptosis following ICH is one of the main causes of inflammation. Chen et al. reported that a ferroptosis-induced inflammatory response in the rat brain after ICH, and Fer-1 treatment significantly reduced ROS and inflammatory cytokines, such as IL-1*β* and TNF-*α*, which indicated that inhibition with Fer-1 alleviated neuroinflammation and improved neural functional recovery [36], which was in agreement with our results. In addition, ferroptosis induced expression levels of prostaglandin-endoperoxide synthase 2 and COX-2. Notably, COX-2 is highly expressed in neurons and is involved in the inflammatory response after ICH, with previous studies indicating that inhibition of reduced ICH-induced secondary brain injury [32]. Therefore, further exploration regarding the regulation of ferroptosis may improve strategies for the treatment of inflammatory diseases.

## 5. Conclusions

Collectively, our data confirmed that PIH alleviated hemorrhage-induced inflammation and ferroptosis by suppressing ROS levels and excessive iron accumulation and ultimately promoted the recovery of neurological function. The results provide a theoretical basis for the clinical use of iron-chelating agents for the treatment of ICH.

## Data Availability

The data used to support the findings of this study are available from the corresponding author upon request.

## Supplementary Materials

Supplementary Materials Figure S1: the cytotoxicity analysis of Erastin and PIH treating with PC12 cells. (A) Cell viability was analyzed by MTT assay with gradient treatment of Erastin. (B) Cell viability was analyzed by MTT assay with gradient treatment of PIH. *n* = 3. ^∗^*P* < 0.05, ^∗∗^*P* < 0.01, ^∗∗∗^*P* < 0.001 versus sham group.
