# Supplementary material for: Pyridoxal Isonicotinoyl Hydrazone Improves Neurological Recovery by Attenuating Ferroptosis and Inflammation in Cerebral Hemorrhagic Mice
Source: Biomed Res Int. 2021 Sep 8;2021:9916328. doi: 10.1155/2021/9916328 (PMC8445720; doi:10.1155/2021/9916328)
Supplement: Supplementary Materials — Figure S1: the cytotoxicity analysis of Erastin and PIH treating with PC12 cells. (A) Cell viability was analyzed by MTT assay with gradient treatment of Erastin. (B) Cell viability was analyzed by MTT assay with gradient treatment of PIH. n = 3. ∗P < 0.05, ∗∗P < 0.01, ∗∗∗P < 0.001 versus sham group. [file 9916328.f1.zip › 9916328.f1/supplementary materials (2).docx]

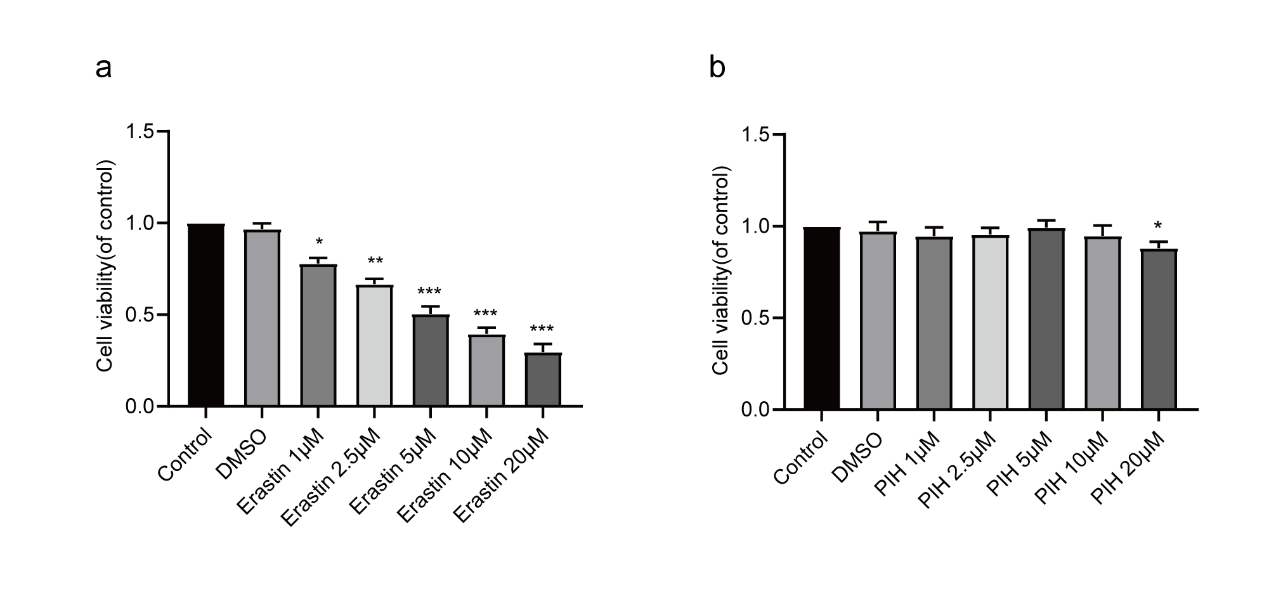
Figure S1: The cytotoxicity analysis of erastin and PIH treating with PC12 cells. (a) Cell viability was analyzed by MTT assay with gradient treatment of erastin; (b) Cell viability was analyzed by MTT assay with gradient treatment of PIH, (F_a_=221.4, F_b_=2.803). n=3. *P<0.05, **P<0.01, ***P<0.001 versus sham group.
